# Reports of Cases Occurring in the Medical Ward of the Buffalo Hospital

**Published:** 1857-08

**Authors:** A. W. Nichols

**Affiliations:** Assistant to Chair of Clinical Medicine, University of Buffalo


ART. II.—*Reports of Cases treated in the Male Medical Ward of the Buffalo Hospital of the Sisters of Charity*, AUSTIN FLINT, M. D., *Attending Physician, during the College Session of* 1856–7, *being from October* 1, 1856 *to February* 18, 1857. Reported by A. W. NICHOLS, M. D., Assistant to Chair of Clinical Medicine, University of Buffalo.

### No. V. *Ecstasy.*

There are two very rare forms of disease involving more or less disturbance of the nervous system, which are presented to our notice in a manner so remarkable, that the diagnosis of them is comparatively easy, although we may never have witnessed previously an instance of either disease. The distinction between *catalepsy* and *ecstasy*, has not always been drawn by writers; yet these diseases differ in important points. Catalepsy and ecstasy occur so rarely, that only thirty-eight cases had been collected by M. Bourdin, in a treatise on this subject, which was published in 1841. It is not stated how many of these cases were instances of ecstasy; but the latter affection appears to be as infrequent as catalepsy, if not more so.

*Catalepsy* derives its name from the peculiar manner in which the patient is attacked, and has been defined to be "a sudden suspension of the action of the senses and of volition; the limbs and trunk preserving the different positions given to them." The disease is paroxysmal, unaccompanied with febrile movement, and usually attended with *complete suspension of the intellectual faculties.* The voluntary muscles retain that condition of contractility in which they were at the moment of the attack; and they appear te possess a tetanic rigidity. But, when the extremities or trunk are handled, they will be found to be *quite elastic;* and we are able, with scarcely any force, to place them in any position we please; and such position will be retained for a greater or less length of time, notwithstanding the postnre which we cause the patient to take, might be extremely inconvenient, and even impossible for him to assume in health. Thus, the patient may be placed so as to rest only on the sacrum, both lower extremities being extended and elevated, and the trunk likewise raised; and this posture, which it is impossible to assume in health, will be maintained for a short time. However, the patient soon manifests a disposition to take a less uncomfortable posture, by slight motion of the extremities and trunk which slowly resume the horizontal position.

Sensibility is almost always null or at least obtuse, during an attack of catalepsy. In regard to those symptoms not immediately referable to the disorder of the nervous system, the skin may be cold, and the joints stiff; the respiration and the circulation scarcely appreciable; such symptoms denote a severe paroxysm; but, in milder attacks, there may be but little variation in the temperature of the surface, or in the elasticity of the articulations; and the respiratory movements and the pulse may be less frequent than in health. The attack may continue for some days, but usually it lasts but a few hours. In a few cases the patients will eat and drink when food and liquids are introduced into the mouth, and digestion will be performed as in health.

Catalepsy is not, of itself, a grave affection, but may become so by complication with other diseases. It would appear to be more frequent among females than males.

There may occur instances in which *the loss of consciousness is incomplete;* these cases are extremely rare. Catalepsy may, therefore, be divided into *complete*, *incomplete* and *complicated.* Those peculiar conditions called *trance* and *daymare*, are instances of incomplete catalepsy. In these cases, the intellectual faculties are not entirely suspended; and the senses are not materially affected; the patient being unable to move or to speak, but conscious of all that is transpiring around him. Such cases, at least, resemble more closely the cataleptic condition than they do that of ecstasy. In some of these instances, the patients present all the appearances of a corpse; and hence are termed cases of *apparent death.*

We desire to direct attention more particularly at this time, to that other condition of the nervous system which very appropriately has been called *ecstasy*, on account of an interesting case of this disease which occurred lately at the hospital.

*Ecstasy* has been defined to be, "that condition in which an individual delivered over entirely to a dominant thought, remains immovable, and a stranger to all that surrounds him." There are present suspension of voluntary movements, and of the action of the senses: this may be complete or incomplete. The patient is absorbed in some idea of the imagination; and his expression depicts the thoughts which so completely engage his mind. At times, this expression is that of ineffable happiness; but always, it is that of *intense abstraction.* Often, there are hallucinations of the senses; and, then, we have that condition which has been called "*vision.*" Dr. Copland states, that many of the Italian Improvisatori are in possession of their peculiar faculty only when experiencing an ecstatic paroxysm. It is not unusual for patients affected with this disease, to sing and to talk. The disease is paroxysmal, and is a purely nervous affection. When the attack comes on, the patient becomes insensible, unmovable, and very rarely the limbs retain, as in catalepsy, the position which they had at the commencement of the paroxysm. His countenance is pallid, and his mouth is partly open; the eyelids are expanded and the eyes are fixed in one direction, usually turned upward. So far is sensibility suspended, that foreign bodies may be applied to the cornea without the patient making any effort to prevent their lodging upon the eye, or to remove them. The pulse and the respiratory movements may be normal, or less frequent than in health; but not notably so. The bowels are usually constipated; and the urine may not be voided, rendering repeated examinations of the bladder necessary. Sometimes when food and drinks are introduced into the mouth, the individual will eat and drink; on the other hand, the food or the liquid having been placed in the mouth, the patient will allow itt o fall or run out, without manifesting any disposition to retain them.

After continuing for a variable period, from a few hours to as many days, the paroxysm terminates, leaving the patient very much dejected, and complaining of fatigue. The attack may recur at periods more or less remote from each other. All writers agree in stating, that ecstasy is an *extremely rare* affection; it is noticed most frequently in persons who meditate long and profoundly on subjects connected with religion; and in those who dwell on the objects of disappointed affection. Grisolle, to whom we are indebted for much of our information on these subjeets, states, that ecstasy sometimes prevails as an epidemic; this appeared to be the case in central Sweden, in the years 1841 and 1842.

It will be remembered that the condition of *trance* was included under the head of *catalepsy*, and that of *vision* under the head of *ecstasy*. The *distinction* between *catalepsy* and *ecstasy* is as follows. In *catalepsy*, there is a *sudden suspension, complete or incomplete, of the intellectual faculties*, of sensibility, of voluntary motion, and of the actions of the senses; the trunk and limbs retaining the position which they had at the time of the attack, and capable of maintaining any position in which we choose to place them, however inconvenient or impossible such a position would be in the interval of the disease: *the paroxysm terminating suddenly*, or the individual recovering in a very short time. *Ecstasy*, although paroxysmal, is *not so sudden in its access; the individual concentrating his intellectual powers upon some imaginary object, is withdrawn more or less rapidly from the influence of external impressions;* the sensibility becomes more and more obtuse, and finally, suspended; the power of voluntary motion is lost; and the expression of the pat'ent shows the profound and the abstracted character of the objects upon which all his intellectual faculties are concentrated; in a variable period of time, the mind breaks loose from the dominant thought; sensibility returns; external impressions are regarded; the voluntary muscles act in obedience to the dictates of the will; and the patient *slowly returns* to a condition of comparative health.

In reference to the *treatment* of ecstasy, but little need be said. That plan of treatment which has been recommended most highly and which has proved most successful, consists chiefly in measures which produce a powerful effect on the mind, rousing the patient to the influence of external impressions, and forcibly drawing away the intellect from the contemplation of the idea which has so long and so firmly held it. A jet of *cold water*, even ice-water, projected upon the face with considerable force, has at times been employed with success. In the following case, the *moral effect* produced by the application of a *heated iron*, proved highly beneficial. If the attack continue for a length of time, it will be necessary, in addition to mental remedies, to administer forcibly the aliment and drink; and to compel the patient to swallow them: the function of digestion being unimpaired in this disease.

Case.* Aug. 3d. Jacob Humble, German, aged 25 years, mechanic. Admitted on the evening of the 1st instant, in a state of unconsciousness. Previous history not ascertained. He was in the hospital during last winter, in a similar condition, which continued for several days.

Yesterday morning I found him apparently in quiet sleep. On raising the lids of eyes, the pupils contracted freely. He was not roused to any manifestation of consciousness. On raising the eyelids, they remained open for some time, and he apparently took no notice. Flies creeping on the face and even on the conjunctiva, did not disturb him. His respiration was perfectly normal; pulse 76, and regular; skin normal; no distension or tenderness of abdomen. Drinks and nutriment taken into the mouth, are retained there for some time, and mostly escape, a small portion being swallowed. He lies motionless; does not change his position, and gives no manifestation of suffering.

The cold douche was applied to the head for some time, but with no effect.

An examination of the precordia revealed no physical sign of heart affection.

An enema of spirits of turpentine and tincture of assafœtida, was alone prescribed.

This morning I find him in the same situation; he has remained so since yesterday. The symptoms are the same as on yesterday. Respiration 16; pulse 76; skin warm and mellow. His countenance and aspect are such as denote perfectly quiet sleep. Deglutition is not impaired; he swallows at certain intervals, and does not manifest difficulty in the act when fluids are introduced into the mouth; but he does not call the act into exercise by any voluntary effort. Applying a bottle of ammonia to the nostrils, occasions contraction of the orbicular muscle of the mouth and flushing of the face, but no manifestation of consciousness nor efforts to escape from the inhalation. No remedy is prescribed to-day; but efforts are to be made to give milk from time to time.

---

* Transcribed from record of case by Prof. Flint. N.

Aug. 4th. No material alteration. He has remained apparently unconscious, and nearly motionless, since last record. He lies now with his eyes opened part of the time, but takes no notice; his eyes are fixed in one direction; winks but seldom. A little erythema and swelling of the right side of the nose. There is no evidence of paralysis, Drinks and nourishment introduced into the mouth, for the most part appear to be ejected. There has been no evacuation of the bowels nor bladder since his admission: the bladder seems to be moderately distended. The catheter was introduced without resistance on his part, or difficulty, and about a quart of water removed; after removing the catheter, a little urine was expelled, together with air. The abdomen is greatly collapsed. On attempting to introduce a little brandy and water into the mouth, he closes the teeth firmly. The pupils are mobile. The fingers may be brought quite in contact with the conjunctiva before he winks; but he occasionally winks when the finger is at some distance from the eye.

After recording the above, whilst engaged in examining other cases, it appeared as if he watched our movements, when he supposed he was unobserved. He turned over on the left side, in bed, which he has not been observed to do before.

No treatment; brandy and nutriment to be administered if practicable.

Aug. 5th. Marked improvement. He lies with his eyes open, and his countenance denotes more intelligence. He does not speak nor appear to heed questions. Makes slight and ineffectual efforts to protrude the tongue when requested. He takes his nourishment freely, and apparently with relish. No evacuation from his bowels or bladder since yesterday. Pulse not accelerated, and skin cool. Last night he asked repeatedly for drink, after some tea had been forcibly administered. He said that he did not hear anything. He wept, last night, violently.

Aug. 6th. Aspect and symptoms not so good as on yesterday. Resists introduction of food. Lies with his eyes open, but appears to take no notice. Passed urine in bed, last night; no dejection since admission.

Directed to day, croton oil, gtt. i, every two hours, till operation; and forcible administration of brandy and nourishment.

Aug. 7th. No material alteration since yesterday. The remedy prescribed yesterday — drinks and nourishment — all have to be forcibly administered. He resists by closing the teeth and by holding them in the mouth; but, finally, they are swallowed. He has taken four drops of croton oil; no cathartic operation; no evacuation of urine; the bladder is moderately distended. His aspect is the same as on yesterday. He lies much of the time with his eyes open, but appears to take no notice. Pulse 76, with more volume than on yesterday; respiration normal. From his aspect to-day, and all along, it is difficult to resist the conviction, that the *coma-like condition is simulated.*

After writing the foregoing, and *having made preparations to introduce the catheter*, I had the instrument in my hand and the body of the patient exposed, when *he began to urinate:* at first in small quantity, and with interrupted efforts, manifestly, and then in a full stream until a pint had escaped.

Aug. 8th. No material change. He had a free evacuation from his bowels last evening; he did not pass it in bed, but *disposed his body so as to pass it on the floor. A heated hammer was touched on several points over the spine;* after which he took drink from a cup into the mouth, but held it in the mouth for some time before swallowing it. (The above application was made with a view to the moral effect.) He shrank from the contact of the iron. On applying the iron, heated in warm water, he bore it for a time with considerable patience; but on continuing the application and on stating, *that it was intended to carry the firing over the whole body, he began to make vigorous resistance*, and finally protruded the tongue, and drank brandy and water freely. The firing was then discontinued, with the promise of repeating it and resorting to the red hot iron if marked improvement does not ensue.

Aug. 9th. *Aspect greatly improved.* The improvement under the circumstances stated, is presumed to show that the previous condition was due, in a great measure, to mental perverseness. Now, he takes notice; drinks without effort or difficulty; had free movement from the bowels, and manifested his wish for that purpose. He does not speak; protrudes the tongue when requested; pulse 76. Brandy and nourishment; no other measures.

Aug. 10th. Aspect improved. Takes his nourishment and drinks freely. *Replies to my questions to-day;* says he is better; says he has a little pain in the epigastrium. *In connection with the recovery of speech*, I should note, that *yesterday I took pains to state that if he did not recover it by morning I should resort to severe firing.*

Aug. 11th. Improving. I have directed that he is to sit up before to-morrow, with the *prospect of firing* if he does not succeed. His condition to-day is as follows: he lies with his eyes open and takes some notice; does not speak of his own accord; makes signs when he wishes to urinate; replies to questions in a feeble whisper, and very slowly. He appears to take very little notice. A fan is given him to keep the flies from his face; he permits them to gather in considerable numbers and then makes a feeble effort to drive them off. No febrile movement; tongue normal. The other day when the firing was resorted to, he used pretty vigorous muscular efforts to escape from the hot iron, and employed his hands for that purpose.

Aug. 12th. This patient is sitting up. He was got up by the attendants, making little or no effort on his own part. He sits in the same position; his eyes fixed in one direction; the lips somewhat separated. Skin cool; pulse slow, and respiration normal. He protrudes his tongue when requested, partially and slowly. He does not reply when addressed. There is considerable difficulty in giving nourishment; he takes drinks only without reluctance. His expression of countenance wears the aspect of the deepest dejection. He observes my movements in the ward, evidently, although he appears to take no notice; but he does not turn his head to watch me as I move to different parts of the ward.

I have directed, that by to-morrow, he must take solid food and masticate it. He has had no dejection; passes urine, and beckoned to the attendant to give him the vessel for that purpose.

Aug. 13th. No material alteration in aspect or symptoms. He has taken a little solid food. Directed him to be carried on to the balcony. He made no effort to walk, *remaining perfectly passive, until threatened with the heated iron.* His mouth remains a little open and saliva flows out.

Aug. 14th. *Marked change.* Expression entirely altered. Takes notice. Greets me with a smile and offers his hand. The improvement began to be manifested yesterday afternoon. Walks of his own accord and with a little aid; and takes his nourishment.

Aug. 16th. Yesterday, this patient was up, dressed, and advanced to meet me, extending his hand. In the afternoon, he again became indifferent, apparently, to external objects, remaining perfectly quiet; his eyes directed to the same object; and refusing nourishment. He remains in this condition this morning. Lies in bed with his eyes open, and takes no notice. Protrudes the tongue slightly when requested; but does not speak. Pulse not accelerated; skin cool. Resorted to *firing over the chest.*

Aug. 18th. This patient remains in the same condition as on the 16th inst. He lies motionless, occasionally turning his head; but rarely, if ever, changing the position of his body. This morning I found him with the eyes open, presenting constantly the same direction, with an expression of vacuity, winking but seldom. This afternoon, they are nearly closed, the lids constantly nictating. He expresses no desires, and appears not to take the least notice of surrounding objects or persons. *Flies* creeping over the face, lips, and even *upon the conjunctiva, seem to occasion no annoyance.* He has had no dejection since the relapse. He urinated on the 16th, not since, as has been observed. He may have urinated in the bathing-tub when undergoing the cold douche. There is no appearance of distension of the bladder, and the abdomen is collapsed. Rough examination of the abdomen produces no apparent sensation. The respiration and pulse are normal. The skin is warm this evening. He has declined drink and nourishment, by resisting the introduction of any thing into the mouth, and holding it in the mouth, and allowing it to escape, until the cold douche or burning is resorted to. Such measures being used solely with a view to their moral effect. Whilst they are employed, he manifests discomfort by writhings of the body, and exclamations of "Gott, Gott;" afterward, for a time. he takes what is given him. This afternoon, on being requested, he partially protruded the tongue. Yesterday and to-day, I have noticed the pupils to be somewhat dilated. They contract freely on exposure to light.

Aug. 26. Patient is sitting up; has an appearance of *intense abstraction.* The expression of the face is indescribable; although it is that of perfect health, yet it shows the profound occupation of the intellectual faculties in some object of the imagination; the term above employed describes as well as can be, the peculiarity. He does not speak; takes food only on compulsion; protrudes the tongue, partially, on being requested. He urinated night before last, indicating the wish by signs; but he has not been observed to urinate since. Has had no dejection since the relapse. No medicine, except an enema; and firing, if he refuses to take nourishment.

Aug. 21st. Sitting up, and presents the same air of intense abstraction. *After firing* this morning, he protrudes the tongue freely, and took solid food. The enema given yesterday, operated well. No treatment but firing.

Aug. 23d. This patient sits up, dressed, and takes food without difficulty; but he still maintains the same condition as regards silence, motionless position, and countenance of abstraction. The attendant leads him about the ward; he walks slowly, with his eyes fixed on the ground, like a somnambulist. The firing is continued, and it is apparently by this means that he is made to eat and to walk about.

The experiment is now being tried, of *leaving him unsupported,* standing in the center of the ward. (When placed near the door or bed, he slowly extends the hand and supports himself.) He remains perfectly motionless. After standing about ten minutes, he gradually inclined to one side, and *finally dropped down, but evidently in a way not to receive injury.* He managed the fall so as to strike *first the nates,* and protected the head by throwing out the hands. When placed in a particular attitude, resting against a bedstead, he remains in the same position as long as his strength permits, and then he gradually slides down. He gives not the least sign of observation or interest in persons or things around him; but he *provides for self preservation in falling.* He is led slowly throughout the ward by the attendant taking hold of the ends of the fingers.

Aug. 25th. Yesterday, this patient was somewhat improved: he talked, and he walked about of his own accord; and his expression was totally changed. He ate and drank readily. To-day, he is silent, and takes food with reluctance. *Firing* is to be resumed; and he is to be made to walk in the open air. He is sitting up; and he has the same fixed expression as heretofore.

Aug. 26th. This morning, Jacob *walks about, eats, and talks freely.* His expression is totally changed. Says he has not had a dejection for a long time, and wishes something to open the bowels.

Aug. 28th. Up and dressed all day; walks out of doors; eats well and talks freely. He seems feeble both as respects body and mind. Walks slowly, and talks as if the mind acted slowly. His expression has entirely changed; but he is still disposed to keep his eyes fixed on one object, and presents, except when addressed, a contemplative aspect. He complains of some pain in the abdomen, and says he has a little headache.

I cannot obtain much information respecting the state of his mind before and during the state of ecstasy. He says, in reply to my question, that he was not happy before being ill; but is more so now. He said, to a German patient in the ward, that he had singing in the ears, and this took away his mind; that he recollected most that had occurred during his illness; and that he could not open his mouth to eat; and that "God above could cure him, and He brought about his illness." From another German patient, I learned, that Jacob told him, he had been *crossed in love*, and that this was the occasion of his illness. I can find no evidence that the attack was due to religious excitement.

Sept. 23d. From date of last record to the present time, this patient has been steadily and rapidly improving; exercising daily in the open air; taking his food and drink readily; answering questions; and his expression being quite cheerful.

To-day he leaves the hospital, reporting quite well; and presenting a healthy and cheerful appearance.

